# Percutaneous Coronary Intervention Difficulty in Tortuous Coronary Anatomy: A Case Report

**DOI:** 10.7759/cureus.89382

**Published:** 2025-08-04

**Authors:** Ahmad Jalil, Fatima Rajab, Mehnaz Nadeem, Eisha Mazhar

**Affiliations:** 1 Internal Medicine, Baptist Memorial Hospital-North Mississippi, Oxford, USA; 2 Internal Medicine, King Edward Medical University, Lahore, PAK; 3 Internal Medicine, Shifa International Hospital Islamabad, Islamabad, PAK

**Keywords:** coronary artery angiography, coronary artery tortuosity, interventional and structural cardiology, primary percutaneous coronary intervention (pci), st-elevation myocardial infarction (stemi), stent delivery failure

## Abstract

One of the relatively common anatomical variants of coronary vessels that is often overlooked in clinical practice is coronary artery tortuosity (CAT). CAT can have a significant impact on coronary blood flow and procedural outcomes during percutaneous coronary intervention (PCI). It is defined by bends, curves, or loops within the coronary vasculature that can lead to increased vascular resistance. These structural changes can impair myocardial perfusion even in the absence of obstructive coronary disease. From an interventional perspective, tortuosity can present considerable technical difficulties, particularly in advancing guidewires, balloons, and stents through the affected segments.

We describe the case of a patient whose PCI was complicated by extensive tortuosity involving the right coronary artery. She had high amounts of thrombosis that were impacting the blood flow, but the tortuosity of her vessel was making it hard to make the lumen patent again. Multiple attempts had to be made before adequate blood flow was achieved.

This case highlights the often-underestimated impact of CAT on both coronary perfusion and interventional success. Recognizing this anatomical feature early and adapting the procedural strategy accordingly can significantly improve the chances of success and reduce the risk of complications in patients undergoing PCI.

## Introduction

Coronary artery tortuosity (CAT) is a common angiographic finding, particularly observed in older individuals and those with comorbid conditions such as hypertension, atherosclerosis, and connective tissue disorders. Evidence suggests a potential role of elastin degradation in the development of tortuosity, as elastin is a key structural component of the extracellular matrix that provides elasticity to vascular walls [1].

While often dismissed as a benign anatomical variant, coronary tortuosity carries significant clinical implications. One of the major concerns arises during percutaneous coronary intervention (PCI), where excessive vessel curvature can pose increased technical difficulties. These anatomical challenges are associated with lower procedural success rates, higher incidences of target vessel failure, and increased risk of stent thrombosis [2,3]. Notably, stent delivery failure occurs in approximately four percent of all PCIs, and in more than 90% of these cases, the failure is attributed to complex vascular anatomy, primarily severe tortuosity or extensive calcification [4].

Beyond procedural hurdles, the hemodynamic consequences of tortuosity are also clinically relevant. The altered flow patterns are characterized by increased resistance and turbulent flow and can compromise myocardial perfusion, potentially leading to ischemia even in the absence of significant atherosclerotic stenosis [5].

In this report, we present the case of a female patient who experienced ST elevation myocardial infarction (STEMI) due to thrombosis in the setting of significant right coronary artery (RCA) tortuosity. The tortuous anatomy directly contributed to both the ischemic event and the technical difficulty encountered during PCI. We also explore and discuss various strategies that may increase the likelihood of a successful outcome in such challenging anatomical settings.

Through this case report, we aim to highlight the ongoing need for further research to optimize PCI strategies in patients with CAT. Additionally, we seek to provide a concise overview of the currently available treatment modalities, summarizing them clearly and effectively to facilitate clinical decision-making.

## Case presentation

A 49-year-old woman with a prior medical history significant for coronary artery disease (CAD), hypertension, hyperlipidemia, and previous PCI to her RCA in 2023 and left circumflex artery in 2019 arrived at the Emergency Room with sudden, intense chest pain. Following several days of exhaustion, she experienced mild dyspnea and two to three hours of substernal, pressure-like chest pain that spread to her left arm. Although she had questionable compliance with medications and medical follow-up in the past, she had recently been compliant with aspirin and clopidogrel. Her initial labs are presented in Table 1.

**Table 1:** Initial lab values of the patient including a complete metabolic profile and lipid panel

Lab Test	Value	Normal Range
Sodium	141	135-145 mmol/L
Potassium	3.8	3.5-5.0 mmol/L
Chloride	104	98-107 mmol/L
Carbon Dioxide	26	22-29 mmol/L
Anion Gap	11	8-16 mmol/L
Glucose	124 (High)	70-100 mg/dL
BUN	15	7-20 mg/dL
Creatinine	0.89	0.60-1.20 mg/dL
BUN/Creatinine Ratio	16.9 (High)	10-15
Calcium	9.0	8.6-10.2 mg/dL
Protein Total	7.9	6.0-8.3 g/dL
Albumin	3.5	3.4-5.4 g/dL
Bilirubin Total	0.2	0.1-1.2 mg/dL
AST	20	10-40 U/L
ALT	20	7-56 U/L
ALP	109	44-147 U/L
eGFR 2021 CKD-EPI-cr	79.6	>90 mL/min/1.73 m²
Triglycerides	99	<150 mg/dL
Cholesterol	173	<200 mg/dL
HDL	37 (Low)	>40 mg/dL (men), >50 mg/dL (women)
Cholesterol/HDL Ratio	4.7	<5.0
LDL Calculated	116 (High)	<100 mg/dL optimal

**Figure 1:**
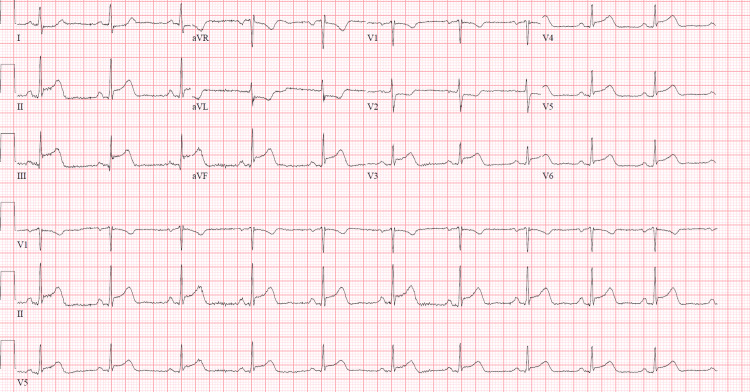
EKG showing inferior wall MI with ST elevations in II, III, and aVF

She was hemodynamically stable and not experiencing any severe distress when she was brought by the EMS. No significant finding was noticed on the physical exam other than severe chest pain. An acute inferior STEMI was confirmed by the initial EKG, which showed ST-segment elevations in leads II, III, and aVF with reciprocal depressions in aVL, as shown in Figure 1.

Troponins at that time were mildly elevated in 20s, but with her prior history of multiple CAD, current symptoms of typical chest pain, and significant ST elevations on EKG, she was scheduled for emergent catheterization right away, and troponin repeat wasn't deemed necessary. She received aspirin, intravenous unfractionated heparin, and cangrelor right away. She was also brought to the cardiac catheterization lab for an urgent coronary angiography.

**Figure 2:**
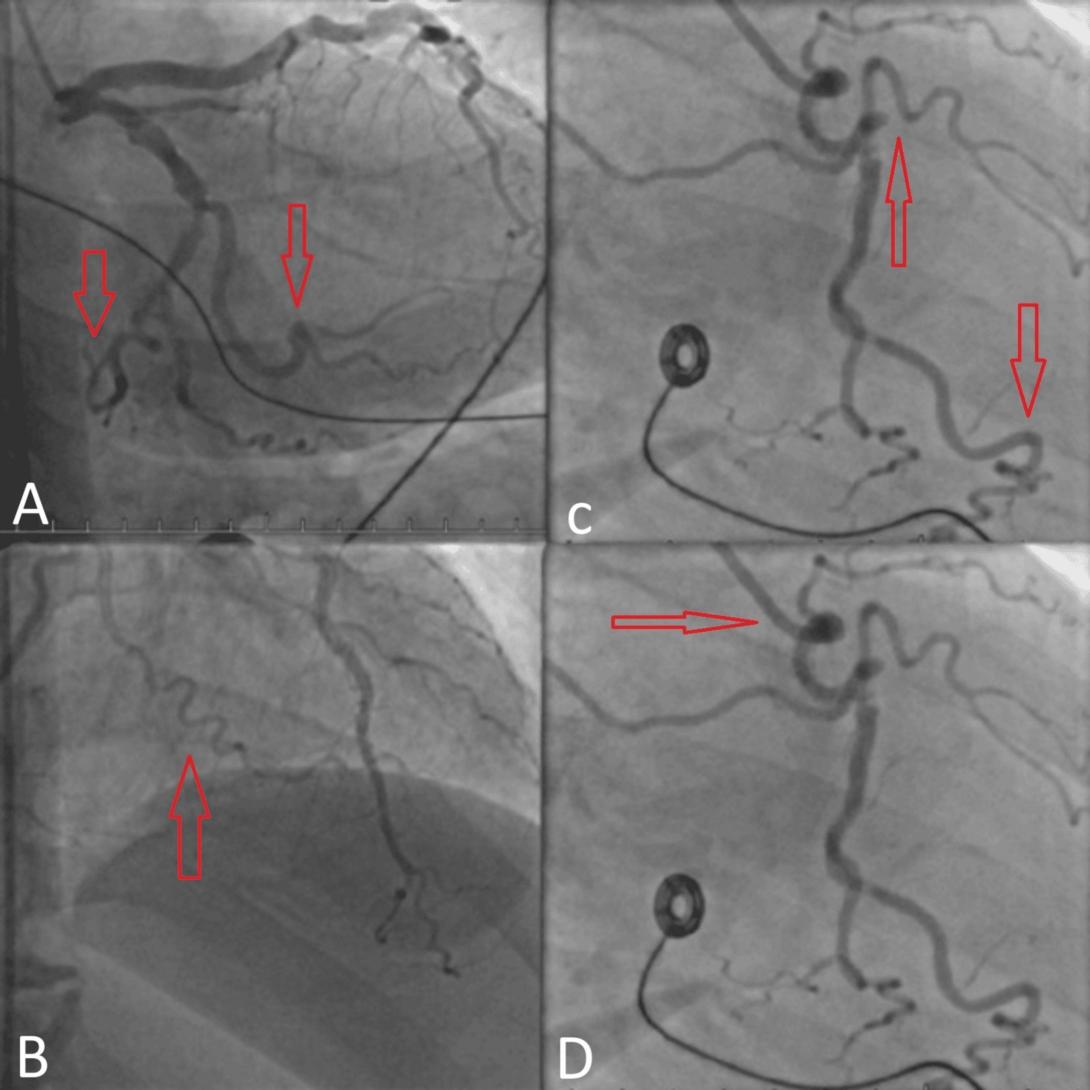
Red arrowheads pointing towards tortuous coronary vessels seen on angiogram. No balloon or microcatheter could be advanced due to excessive tortuosity and resistance present in these vessels

In the catheterization lab, a moderate-caliber but extremely tortuous RCA with previously inserted stents in the proximal and mid-segments was seen during angiography. There was evidence of stent malposition in the tortuous segment and a new 80% thrombotic stenosis in the proximal RCA. Initially, a JR4 guide catheter was used to access the RCA, and a BMW guidewire was passed through the lesion successfully. However, no balloon or microcatheter could be advanced, and all attempts at wire passes ended in sub-stent tracking due to excessive tortuosity and resistance. The tortuosity of her coronary vessels is shown in Figure 2.

However, following wire manipulation, the patient's chest pain completely subsided, the TIMI-three flow spontaneously returned, and the ST segments on the ECG returned to normal. No additional PCI attempts were made due to the clinical stability of the patient as well as technical difficulties. Echo was performed the next day, which showed normal ejection fraction and concentric left ventricular hypertrophy, a finding that was seen in prior echocardiograms as well.

The patient was medically managed with dual antiplatelet therapy, beta-blocker, ACE inhibitor, and high-intensity statin. She remained chest pain-free, was able to ambulate without difficulty, and was discharged with close cardiology follow-up and emphasis on strict medication compliance. In the primary care physician's (PCP’s) office a few days later, she was still chest pain free and was doing well.

## Discussion

The coronary vasculature is structurally unique in that blood flow to the myocardium progresses from the outer epicardial surface inward. Two main types of vessels, resistance and capacitance vessels, work in tandem to ensure effective coronary perfusion. Resistance vessels, which include small arteries and arterioles, respond to metabolic and autonomic signals by adjusting their diameter. This fine-tuned regulation of vascular resistance is what allows the heart to match blood flow with myocardial oxygen demand [6].

Capacitance vessels, on the other hand, mainly venules and coronary veins, act as blood reservoirs. They help buffer changes in volume and modulate venous return to the heart, especially during periods of fluctuating preload or hemodynamic stress [7]. Together, these two systems maintain a delicate balance between myocardial supply and demand, a balance that can easily be disturbed by structural abnormalities.

One such abnormality is CAT, a condition where the arteries twist and elongate more than usual. Since the origin and endpoint of these vessels are anatomically fixed, any excess length forces the arteries to form curves, loops, or sharp bends. Several factors have been linked to the development of CAT, including hypertension, atherosclerosis, chronic mechanical stress (like axial tension), and genetic connective tissue disorders [8-10].

Clinically, tortuosity is often described based on the pattern of vessel curvature; terms like S-shaped, C-shaped, kinking, and looping are commonly used in both diagnostic angiography and research [11]. The physiological burden of tortuous vessels has been explored through computational models. Xie et al. found that coronary resistance can increase by as much as 92% during exertion in tortuous vessels, with diastolic driving pressure rising by up to 96% compared to non-tortuous arteries [12]. These numbers illustrate how significantly tortuosity can interfere with coronary hemodynamics, particularly during exercise, when the heart’s demand for oxygen peaks. Clinically, this may explain why patients with severe CAT often present with angina or ischemia, even when there’s no visible obstruction on angiography. In fact, CAT is increasingly recognized as an independent predictor of non-obstructive coronary artery disease [13].

From an interventional cardiology standpoint, tortuosity complicates PCI in several ways. It can make it harder to advance wires, balloons, or stents, and may increase the risk of complications like wire prolapse, stent malposition, or failure to cross the lesion altogether. Tortuosity is also often seen in patients with spontaneous coronary artery dissection and those with systemic vascular conditions like fibromuscular dysplasia, suggesting a broader vascular vulnerability [2,14]. Even though CAT is common, its long-term impact on outcomes remains understudied, and we still don’t fully understand how best to manage it in the catheterization lab [1,15].

Esenboğa et al. [16] looked at how CAT affects myocardial perfusion using the TIMI frame count (TFC) and myocardial blush grade (MBG), two well-established angiographic measures. They found that tortuous vessels, including the RCA, LAD, and circumflex, consistently had higher TFC and lower MBG compared to non-tortuous vessels. This suggests that CAT not only delays epicardial blood flow but also impairs downstream microvascular perfusion. Interestingly, even in cases where MBG was preserved, TFC remained elevated, indicating that tortuosity itself may independently compromise coronary flow.

In our patient, PCI was complicated by tortuous coronary anatomy, requiring adjustments in both technique and equipment. As described by Chawla et al. [17], initial support was enhanced using larger guide catheters such as Amplatz or Extra Backup. When passive support was insufficient, active techniques like deep seating and controlled rotational maneuvers were employed. Additional strategies such as the buddy wire, anchor wire, and anchor balloon techniques helped straighten the vessel and stabilize equipment. In challenging cases, the mother-child approach or guide catheter extension devices enabled deeper intubation for improved backup. Lesion preparation with specialty balloons or atherectomy devices facilitated device delivery, while intravascular imaging and physiological assessment guided precise treatment. The combined use of these techniques allowed for safer and more effective intervention in this complex anatomy.

As far as the prevalence of CAT goes, it varies depending on the population studied and the definition used. In a large angiographic study of patients with suspected CAD, coronary tortuosity was present in 39.1% of patients, with a higher prevalence in women and those with hypertension [18]. In a cohort of postmenopausal women with non-obstructive CAD, the prevalence was 47% [19].

In summary, CAT presents both physiological and procedural challenges. As highlighted by our case, recognizing tortuosity early and adjusting your strategy accordingly, both in terms of planning and execution, can be the key to a successful outcome. Given how common CAT is, and how often it complicates PCI, we need to continue exploring its clinical implications and develop standardized approaches for managing it in the catheterization lab.

## Conclusions

CAT is an anatomical variation of the coronary blood vessels that, on the one hand, can induce ischemia through interruption of blood supply to the heart and, on the other hand, make PCI and stent placement very difficult and dangerous for the patient. Through our case, we aim to highlight the continuous need for advancements in cardiac procedures and equipment to deal with this, especially in patients with high clot burdens who need angioplasty, as well as shed light on some of the current techniques that could be employed. A better understanding of the pathophysiological and mechanical impact of CAT is essential to improving outcomes in patients with this increasingly recognized anatomical variant. Early recognition, appropriate imaging, and the use of tailored interventional strategies could potentially be lifesaving in patients having CAD.

Limitations of our case report include its single-patient nature, the relatively recent occurrence of the event, and the absence of extensive longitudinal follow-up. Additionally, our literature review highlights an ongoing lack of consensus regarding the optimal hierarchy or standardized techniques for percutaneous coronary intervention in patients with CAT. Consequently, further research involving larger patient cohorts and more comprehensive data collection will be crucial to developing clear, evidence-based strategies for managing PCI in patients with CAT.
